# Deep genetic divergences among Indo-Pacific populations of the coral reef sponge *Leucetta chagosensis *(Leucettidae): Founder effects, vicariance, or both?

**DOI:** 10.1186/1471-2148-8-24

**Published:** 2008-01-26

**Authors:** Gert Wörheide, Laura S Epp, Luciana Macis

**Affiliations:** 1 Courant Research Center Geobiology, Georg-August-Universität Göttingen, Goldschmidtstr. 3, D-37077 Göttingen, Germany; 2 Institut für Biochemie und Biologie, Evolutionsbiologie/Spezielle Zoologie, Universität Potsdam, Karl-Liebknecht-Str. 24-25, D-14476 Potsdam, Germany

## Abstract

**Background:**

An increasing number of studies demonstrate that genetic differentiation and speciation in the sea occur over much smaller spatial scales than previously appreciated given the wide distribution range of many morphologically defined coral reef invertebrate species and the presumed dispersal-enhancing qualities of ocean currents. However, knowledge about the processes that lead to population divergence and speciation is often lacking despite being essential for the understanding, conservation, and management of marine biodiversity. Sponges, a highly diverse, ecologically and economically important reef-invertebrate taxon, exhibit spatial trends in the Indo-West Pacific that are not universally reflected in other marine phyla. So far, however, processes generating those unexpected patterns are not understood.

**Results:**

We unraveled the phylogeographic structure of the widespread Indo-Pacific coral reef sponge *Leucetta chagosensis *across its known geographic range using two nuclear markers: the rDNA internal transcribed spacers (ITS 1&2) and a fragment of the 28S gene, as well as the second intron of the *ATP synthetase beta subunit*-gene (*ATPSb*-iII). This enabled the detection of several deeply divergent clades congruent over both loci, one containing specimens from the Indian Ocean (Red Sea and Maldives), another one from the Philippines, and two other large and substructured NW Pacific and SW Pacific clades with an area of overlap in the Great Barrier Reef/Coral Sea. Reciprocally monophyletic populations were observed from the Philippines, Red Sea, Maldives, Japan, Samoa, and Polynesia, demonstrating long-standing isolation. Populations along the South Equatorial Current in the south-western Pacific showed isolation-by-distance effects. Overall, the results pointed towards stepping-stone dispersal with some putative long-distance exchange, consistent with expectations from low dispersal capabilities.

**Conclusion:**

We argue that both founder and vicariance events during the late Pliocene and Pleistocene were responsible to varying degrees for generating the deep phylogeographic structure. This structure was perpetuated largely as a result of the life history of *L. chagosensis*, resulting in high levels of regional isolation. Reciprocally monophyletic populations constitute putative sibling (cryptic) species, while population para- and polyphyly may indicate incipient speciation processes. The genetic diversity and biodiversity of tropical Indo-Pacific sponges appears to be substantially underestimated since the high level of genetic divergence is not necessarily manifested at the morphological level.

## Background

Knowledge about the processes that generate and maintain marine biodiversity, and the evolutionary relationships and genetic variation of regional populations, along with assessments of the amount of demographic connection between these populations, are essential for understanding and effectively conserving and managing marine resources [1] such as the highly diverse coral reefs [2]. However, this information is currently lacking in the Indo-Pacific for most coral reef organisms other than fish and corals [3], which is surprising considering their diversity and the significant ecological and economic roles played by this ecosystem [4].

Because marine organisms frequently have high dispersal potential (e.g. [5]), their ranges have often been considered to be vast. However, populations have frequently been found to be highly genetically differentiated [6]. Sometimes they exhibit fine-scale endemism [7], and (cryptic) speciation is a common process in the sea [8]. Two main processes have been invoked as responsible for allopatric speciation in the tropical Indo-West Pacific (IWP): vicariance, where a species' previously coherent geographic range has become fragmented following the formation of a barrier to dispersal; or speciation through a founder effect, where a new population is established by a small number of individuals, often by long-distance dispersal, and subsequent restricted gene flow has led to speciation (reviewed in [9]). Both processes are probably the extremes of a continuum rather than being mutually exclusive [10], but the degree of their interplay remains poorly understood.

This study focuses on tropical marine sponges, a highly diverse, ecologically and economically important, but notoriously understudied, marine invertebrate taxon [11]. Biodiversity analyses of Australasian tropical sponges based on species occurrence data have, for example, revealed quite different trends from those of other marine phyla in the IWP [12]. Latitudinal gradients in sponge diversity were not evident, but various environmental factors were prominent at small spatial (α-diversity) scales. Patterns observed at larger spatial (γ and ε) scales of diversity have been ascribed to biogeographic factors and connectivity (reviewed in [13]). However, investigations based on morphometric data alone did not suffice to unravel the biogeographic factors and connectivity among populations.

The lemon-yellow calcareous sponge *Leucetta chagosensis *Dendy 1913 (Porifera: Calcarea: Leucettidae) served here as a model to test biogeographic and phylogeographic hypotheses, and to determine the degree of connectivity vs. isolation among populations, with a focus on the SW Pacific. *L. chagosensis *has a wide Indo-Pacific distribution, ranging from the northern Red Sea to the central Pacific (Moorea, Tuamotu) and from Okinawa (Japan) to Brisbane (Moreton Bay, Australia), and was considered to be a single species throughout this range [14]. However, the validity of this assumption has been challenged by molecular data [15]. *L. chagosensis *is relatively common and is typically found in shaded habitats below a water depth of 15 m. It is often the dominant macro-sponge in semi-cryptic habitats, e.g., on the Great Astrolabe Reef (Fiji) (Wörheide, pers. observ.). It is viviparous, and as such, is thought to have low dispersal capabilities, as is the case for many other sponges [16]. Initial attempts to use mitochondrial gene sequences (cytochrome oxidase II) to resolve phylogeographic patterns of *L. chagosensis *in the western Pacific were unsuccessful due to the low variability of this marker [17]. Such low intraspecific mtDNA variation is apparently a general mtDNA feature in sponges and anthozoan cnidarians, presumably due to the low rate of their mtDNA evolution [18-20]. A subsequent phylogeographic study, focussing on the SW Pacific, used rDNA internal transcribed spacer (ITS) sequences that provided better resolution and uncovered a deep phylogeographic break on the Great Barrier Reef with distinct northern and southern clades. Each of these GBR clades was more closely related to the Indonesian clade than to each other, and a vicariance scenario of fragmentation and range expansion during and after the last glacial sea level low was suggested as a cause for this structure [15].

The aims of the present study were as follows: to unravel the phylogeographic structure of *L. chagosensis *over its full geographic range using two nuclear loci; to estimate the degree of connectivity among regional populations; to determine whether vicariance or founder dispersal was responsible for generating the observed patterns; and to test *L. chagosensis*' taxonomic status as a widespread Indo-Pacific species.

## Results

### rDNA

#### ITS, partial 28S sequences

**Figure 1:**
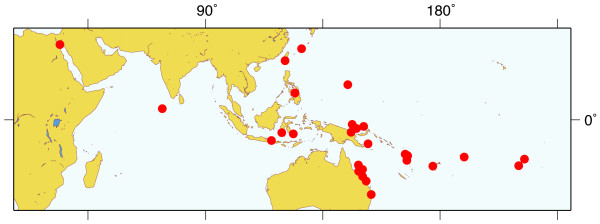
**Geographical map showing the location of collection sites**. Distribution of collection sites across the Indo-Pacific, covering all of the known geographic range of *Leucetta chagosensis*. Map was generated using Map-It [92].

Our data set of 176 individuals [see Additional file 1] includes samples covering the known geographic extent of *L. chagosensis *(Fig. 1). The first rDNA fragment used (referred to hereafter as ITS) includes the 3' end of the 18S gene, the full ITS1, the 5.8S gene, and the nearly complete ITS2. It was combined with a fragment from the C2-D2 region of the 28S gene. See Table 1 for general features of both fragments. Although previous studies did not detect any intragenomic polymorphisms (IGPs) in the ITS regions either by SSCP [15] or by the subcloning of amplicons [21], a few IGPs were detected here, both in the ITS and partial 28S gene. All polymorphic sequence types could be resolved into two sequence types per individual. Both rDNA fragments had a high GC content (Table 1, 53–61.9%), in the normal range for sponge rDNA [21,22].

**Table 1:** Features of sequenced regions.

	aligned length (bp)	GC (%)	max. p-distance (%)	**nucleotide diversity (**π**)**
**ITS rDNA**	743	53	2.16	0.00863
**28S rDNA**	306	61.9	2.63	0.0066
***ATPSb*-iII**	1089	40.2	9.57	0.03524

Maximum aligned length (including indels), GC content, maximum uncorrected p-distance in percent, and nucleotide diversity of each sequenced fragment. ITS rDNA: ITS1 and ITS2 plus flanking regions; 28S rDNA: C2-D2 region of the 28S rDNA; *ATPSb*-iII: second intron of the *ATP synthetase beta subunit *gene.

#### rDNA sequence type network

**Figure 2:**
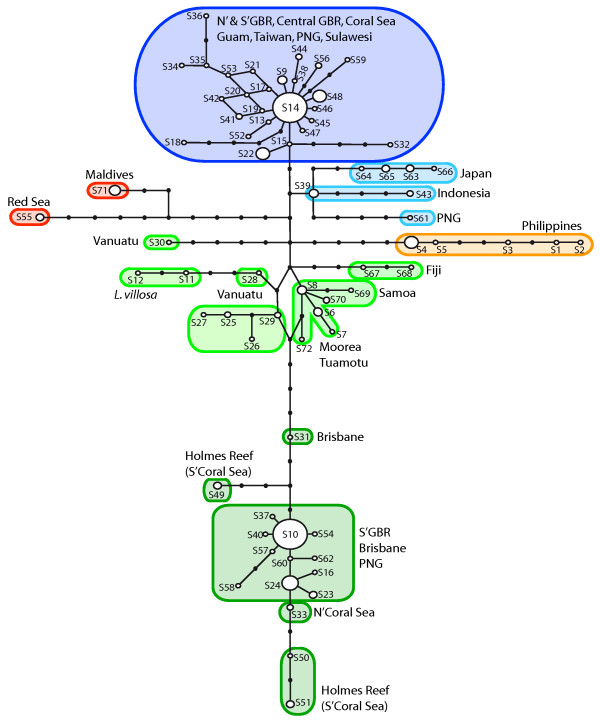
**Unrooted network of 72 unique rDNA sequence types**. Sequence type network constructed from the concatenated ITS and 28S (C2-D2 region) alignment using statistical parsimony. Numbers with the prefix "S" indicate sequence type number [compare with Additional Table 1] and small black dots denote hypothetical (not observed) intermediate sequence types. The size of the white circles indicates the relative frequency of the sequence type in the data set. Large clades are color coded according to their main geographic distribution. Blue: Northwest Pacific; green: Southern Pacific; orange: Philippines; red: Indian Ocean.

The estimated statistical parsimony network, which had a maximum calculated connection limit of 14 steps (at 95% confidence), is shown in Figure 2. It was congruent in its general topology with a phylogeny estimated by Bayesian inference (BI), which had low branch support (similar to [15]) (not shown). The network showed the sequence types oriented along a central axis, with two main clusters near the top and bottom ends. The top cluster, centered around the most frequent sequence type S14, contained specimens from the Northern GBR, the Coral Sea, Guam, Taiwan, Papua New Guinea, and Sulawesi (shaded in blue). The bottom cluster, centered around the second most frequent sequence type S10, contained specimens from the Central and Southern GBR, the Coral Sea, and PNG (shaded in dark green). Sequence types from the Central South Pacific (shaded in lighter green) form an ambiguous loop in the network that could not be resolved with confidence. *Leucetta villosa *Wörheide & Hooper 1999, the putative GBR-sister taxon of *Leucetta chagosensis*, is located within this part of the network. Towards the top cluster, a number of deeply separated branches contain sequence types from the Philippines, the Red Sea, and one specimen from Vanuatu. The last branch, closest to the top cluster, contains sequence types from Japan, Indonesia, and PNG (shaded in lighter blue).

#### Analysis of Molecular Variance (AMOVA)

AMOVAs were carried out for two subsets of the data: subset 1 contained all samples from the total 15 geographically pooled populations in four regional groups (i.e. the whole data set), and subset 2 contained a more spatially restricted set of individuals from the SW Pacific only. For subset 1 (Table 2), the rDNA genetic variation was hierarchically structured, with about 41% distributed among populations within the groups, about 42% within populations, and only about 17% among the four groups. Fixation indices showed significant high genetic structuring at all hierarchical levels, with the highest structuring within populations and the lowest among groups. For subset 2, less than 3% of the variation was distributed between the two groups, with no significant differentiation. Slightly more than a third of the variation was distributed among populations within the groups, which were highly differentiated, and a little less than two thirds of the variation was distributed within highly differentiated populations.

**Table 2:** Results of AMOVA analysis (rDNA sequences).

	Subset 1: 15 populations, 4 groups				Subset 2: SW Pacific only			
Source of variation	Degrees of freedom	Variance components	% of variation	Fixation indices	Degrees of freedom	Variance components	% of variation	Fixation indices

Among groups	3	0.87433	16.89	*φct*: 0.16893 *	1	0.12755	2.92	*φct*: 0.02918
Among populations within groups	11	2.12873	41.13	*φsc*: 0.49489 **	6	1.70404	38.98	*φ*sc: 0.40156 **
Within populations	335	2.17268	41.98	*φst*: 0.58022 **	262	2.53949	58.10	*φst*: 0.41902 **

Hierarchical structuring of molecular variation (AMOVA) within and among *a priori *defined populations and groups based on rDNA sequences only. Subset 1: 15 populations grouped into four groups; Subset 2: 8 populations from the SW Pacific grouped into two groups. Statistical significance is based on 10,100 permutations in Arlequin: * *P *< 0.05; ** *P *< 0.001.

### *ATP-Synthetase beta subunit* intron II (*ATPSb*-iII)

#### Sequences and alignment

Both alleles of *ATPSb*-iII, a phase-0 intron [see Additional file 2], could be resolved from 113 specimens of *L. chagosensis*. The length of the intron varied between about 750 bp in most individuals to 1089 bp in the population from Okinawa, Japan, due to a very long insertion. The maximum uncorrected p-distance among alleles was 9.57%, with a nucleotide diversity (π) of 0.03524 – about a four-fold increase in variation compared to the rDNA regions sequenced (see Table 1). No significant deviation from neutrality was found (Tajima's D: -1.20920, not significant) and no recombination was detected.

Several indels of various lengths were observed; some were minisatellite repeats restricted to certain populations, e.g. Vanuatu. Length variant heterozygotes differed either in the number of residues in a stretch of homomer thymidines starting at position 301 (max T_10_) or in a few private indels. After collapsing the 226 alleles of the 113 specimens sequenced, 89 unique alleles remained.

#### Phylogeny estimation

**Figure 3:**
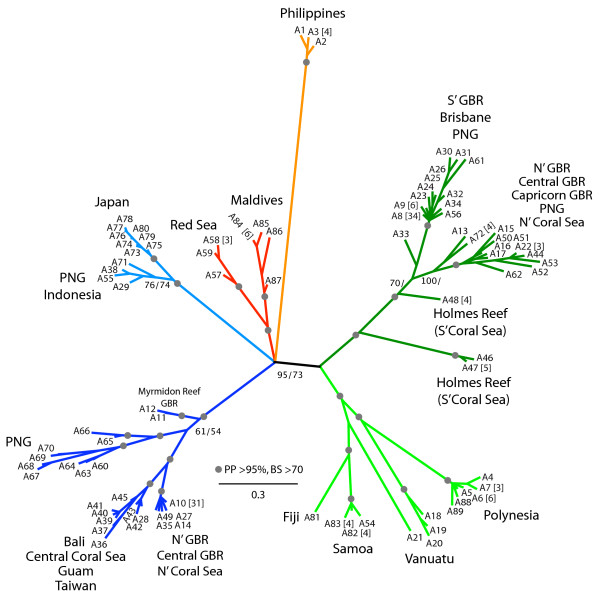
**Unrooted phylogeny of 89 unique *ATPSb*-iII alleles**. Unrooted Bayesian phylogeny where highly supported branches with >95% posterior probability and >70% Maximum Likelihood non-parametric bootstrap proportions are indicated by a grey circle; numbers at branches are given if values were less than the above (main clades only). See Additional Figure 3 for detailed values. Numbers with an "A" prefix indicate allele number [compare with Additional Table 1]. Numbers in square brackets after allele numbers indicate how many individuals carried that allele, if more than one. Color code of larger clades is the same as in Figures 2 and 4.

The estimated unrooted Bayesian phylogeny of the 89 unique alleles is shown in Figure 3 [see also Additional file 3]. An estimated phylogeny using the maximum likelihood optimality criterion showed the same topology, and a statistical parsimony network, constructed using TCS with the 95% connection limit, yielded several disconnected networks (not shown). In the Bayesian phylogeny, six main larger clades were present, all of which were well supported by posterior probabilities (PP, >95%) or bootstrap proportions in Maximum Likelihood analyses (BP, >70). The unrooted tree was characterized by one polytomy, in which the relationships among the four main clades could not be resolved. The general phylogeographic patterns were congruent with the rDNA network (Fig. 2). Several populations were reciprocally monophyletic (Red Sea, Maldives, Philippines, Japan, Samoa, Polynesia), while others were paraphyletic (e.g. Vanuatu) or polyphyletic (Papua New Guinea, Great Barrier Reef, Coral Sea). Alleles from the Philippines were most divergent.

An estimated phylogeny with all 226 alleles had the same topology, and showed that alleles from heterozygous individuals that were subcloned to resolve length variant alleles were found in the same larger clade and were not dispersed among distantly related clades (not shown).

#### Analysis of Molecular Variance (AMOVA)

AMOVAs were carried out for two subsets of the data, as defined above for the rDNA. For subset 1 (Table 3), genetic variation was, in part, hierarchically structured, with about 9% distributed among the four groups, about 57% among populations within the groups, and about 34% within populations. Fixation indices showed significantly high genetic divergence within populations and among populations within groups, with non-significant among-group differentiation. For subset 2, about 10% of the variation was distributed among the two groups, which were not significantly differentiated. About 46% of the variation was distributed among highly differentiated populations within groups, and about 44% within highly differentiated populations.

**Table 3:** Results of AMOVA analysis (*ATPSb*-iII sequences).

	Subset 1: 15 populations, 4 groups				Subset 2: SW Pacific only			
Source of variation	Degrees of freedom	Variance components	% of variation	Fixation indices	Degrees of freedom	Variance components	% of variation	Fixation indices

Among groups	3	4.11522	8.92	*φct*: 0.08923	1	3.57969	10.15	*φct*: 0.10153
Among populations within groups	11	26.49117	57.44	*φsc*: 0.63065 **	6	16.12770	45.74	*φsc*: 0.50910 **
Within populations	213	15.51508	33.64	*φst*: 0.66360 **	168	15.55098	44.11	*φst*: 0.55894 **

Hierarchical structuring of molecular variation (AMOVA) within and among *a priori *defined populations and groups based on *ATPSb-*iII sequences only. Subset 1: 15 populations grouped into four groups; Subset 2: 8 populations from the SW Pacific grouped into two groups. Statistical significance is based on 10,100 permutations in Arlequin: * *P *< 0.05; ** *P *< 0.001.

#### Pairwise Fixation Indices (F_ST_)

**Figure 4:**
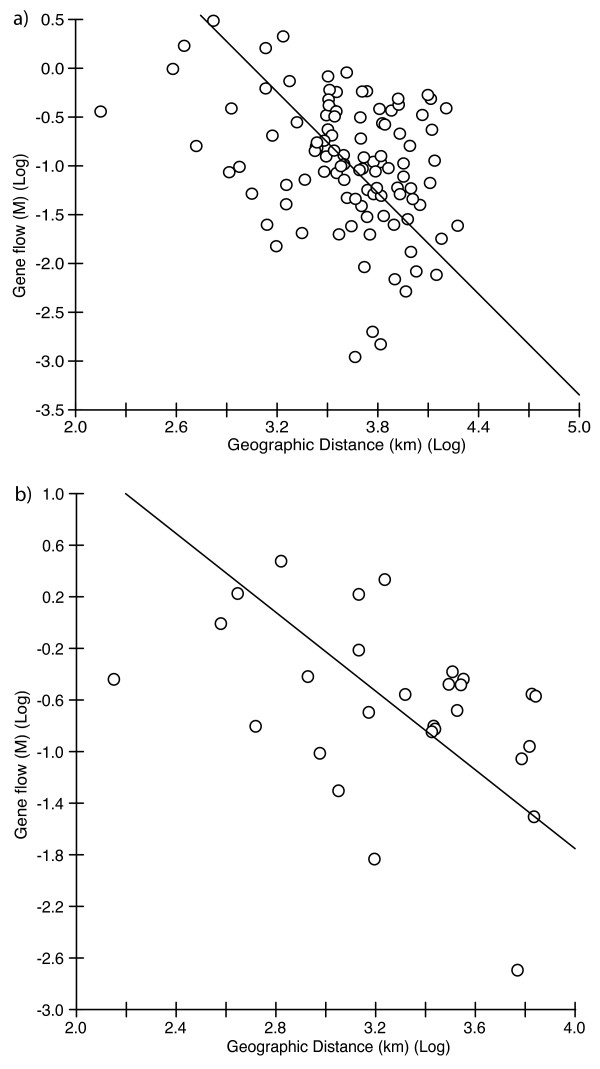
**Graph of relationships between genetic and geographic distances**. a) Relationships between *Leucetta chagosensis *log-transformed gene flow parameters (*M*) and log-transformed distances (km) of the 15 population subset. Thick lines indicate the Reduced Major Axis Regression. Mantel tests revealed a significant major axis regression slope of -0.3451 (*P *= 0.0144). b) The same relationships for the 8 population subset. Mantel test revealed a significant major axis regression slope of -0.4177 (*P *= 0.0332).

All but two pairwise comparisons of genetic differentiation between eight populations in the SW Pacific based on *F*_ST _values were statistically significant at the 99% confidence level (see Table 4). The lowest significant value observed was 0.1, between the population from the Central GBR and Papua New Guinea. Most other pairwise comparisons had values of more than 0.25, and the highest values observed were above 0.9 (0.94: Sunshine Coast/Brisbane vs. Northern GBR; 0.99: Sunshine Coast/Brisbane vs. Polynesia). Only the pairwise comparison between the population from the Capricorn Section of the southern GBR and the adjacent population from the Sunshine Coast and Brisbane indicated no significant genetic differentiation. Testing for a pattern of isolation-by-distance by performing a Mantel test between genetic (*F*_ST_) and spatial distances did not reveal any significant relationships (r = 0.273, *P *= 0.0758) for the whole data set (subset 1: 15 populations), but an analysis of populations along the South Equatorial Current (subset 2: 8 populations) revealed a weak but significant correlation (r = 0.439, *P *= 0.0326) (not shown). However, some populations did not match this trend; as was the case for some of the pairwise comparisons among populations on the East-Australian coast: S'GBR-Central GBR, S'GBR-N'GBR, Brisbane-Central GBR, Brisbane-N'GBR (compare with Table 4). Calculations using log-transformed distances and log-transformed gene flow parameters (*M*) revealed significant negative correlations for the 15 population subset (r = -0.3927, *P *= 0.0144; Fig. 4a) and the eight population subset (r = -0.4177, *P *= 0.0327; Fig. 4b).

**Table 4:** Pairwise *F*_ST _values of eight populations in the SW Pacific (*ATPSb*-iII alleles).

	Northern GBR	Central GBR	Capricorn Section (S'GBR)	Sunshine Coast & Brisbane	Queensland Plateau, Coral Sea	Papua New Guinea (PNG)	Samoa/Fiji/Vanuatu
Northern GBR	-						
Central GBR	0.12*	-					
Capricorn Section (S'GBR)	**0.72**	**0.61**	-				
Sunshine Coast & Brisbane	**0.94**	**0.83**	0.08 ns	-			
Queensland Plateau, Coral Sea	**0.41**	**0.20**	**0.40**	**0.55**	-		
Papua New Guinea (PNG),	**0.29**	**0.10**	**0.47**	**0.63**	**0.13**	-	
Samoa/Fiji/Vanuatu	**0.54**	**0.43**	**0.61**	**0.64**	**0.38**	**0.41**	-
Moorea & Tuamotu (Polynesia)	**0.89**	**0.70**	**0.74**	**0.99**	**0.47**	**0.48**	**0.43**

Pairwise *F*_ST _values of eight populations in the SW Pacific (*ATPSb*-iII alleles). Bold values indicate a significance level of <0.01, * a significance level of <0.05 after 10,100 permutations in Arlequin 3.1. ns = non-significant. Populations correspond to subset 2 of Tables 2 and 3.

### Combined rDNA and *ATPSb*-intron analyses

#### Phylogeny estimation

**Figure 5:**
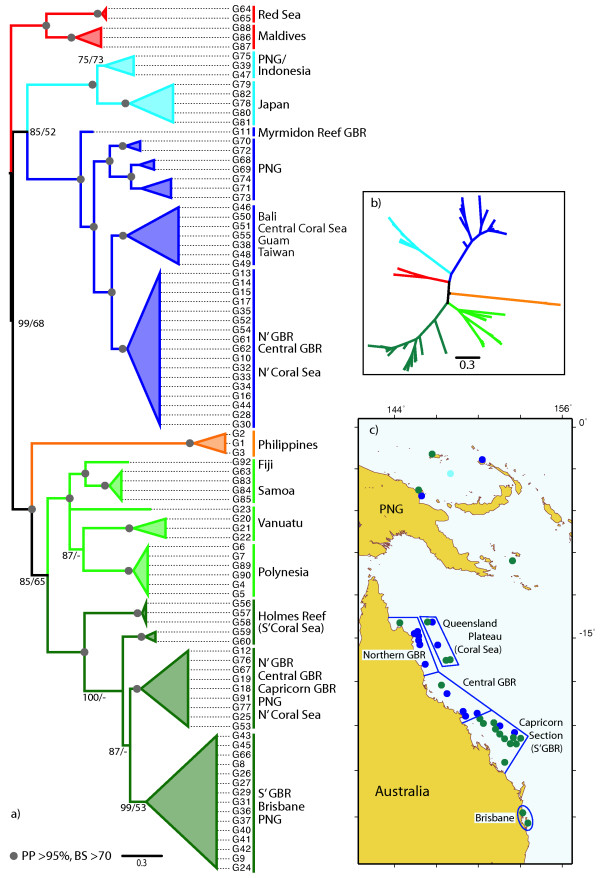
**Phylogeny of 92 genotypes from the concatenated alignment**. a) Bayesian phylogeny of 92 unique genotypes from the concatenated rDNA and *ATPSb*-iII alignment rooted with the populations from the Indian Ocean. Highly supported branches with >95% posterior probability from Bayesian analysis and >70% Maximum Likelihood non-parametric bootstrap proportions are indicated by a grey circle; numbers at branches are given if the values were less than the above. Terminal branches are collapsed for clarity of presentation. See Additional Figure 4 for a fully resolved phylogeny and detailed support values. Numbers with a "G" prefix indicate genotype number (compare with Additional Table 1). Larger clades are color-coded as in Figures 2 and 3. b) Unrooted phylogeny with the same color-coding of clades as in a) showing only the main branches. c) The map displays the area of apparent overlap of two of the deepest diverging clades that have their main geographic distribution in the NW Pacific (blue) and the Southern Pacific (green) in the area of Papua New Guinea and the Great Barrier Reef (GBR). Map also shows subdivision of GBR populations that follows the sections of the Great Barrier Reef Marine Park, as defined for population genetic analyses (see text for details).

All three fragments – ITS, the C2-D2 regions of the rDNA, and *ATPSb*-iII – were obtained for 105 specimens [see Additional file 1]. Among those, 92 unique genotypes were found. The estimated Bayesian phylogeny is shown in Figure 5a. The general topology was congruent with the phylogeny estimated from the *ATPSb*-iII-only alignment. Several deeply diverging major clades were detected, highly supported by posterior probabilities and maximum likelihood bootstrap values, allowing for an increased resolution compared to the *ATPSb*-iII-only phylogeny and solving the polytomy among the four larger clades with their main geographic distribution in the Indo-Northwest Pacific (compared with Fig. 3). A closer relationship of the Indian Ocean clades (Fig. 5b, red) to the remaining ones from the Indo-Northwest Pacific (Fig. 5b, blue) was revealed in an unrooted phylogeny, which also suggested a closer relationship of the Philippine clade with the clades from the southern Pacific [see also Fig. 5a and Additional file 4]. The main clade geographically restricted to the southern Pacific (Fig. 5, green) was, itself, substructured into two sister-groups: the south-central Pacific *sensu stricto *(Fiji, Samoa, Polynesia) and the south-western Pacific, restricted to PNG and Australia's East coast. Several regional populations showed reciprocal monophyly (e.g., the Red Sea, Maldives, Japan, Philippines, Samoa, Polynesia), while others were para- or polyphyletic (e.g., Vanuatu and PNG/GBR). The geographic distribution of two of the main clades (green and blue in Fig. 5a) in the area of the GBR and PNG are also illustrated in Fig. 5c.

#### Neighbor-Net analyses

Neighbor-Net analyses of the two separate rDNA and *ATPSb*-iII partitions of the 92 genotype data set were carried out to explore ambiguities in the data and to evaluate the degree of congruence among both loci [see Additional file 5]. Some ambiguities were detected in the *ATPSb*-iII Neighbor-Net, especially among genotypes from the southern Pacific (small loops in the network). The rDNA Neighbor-Net showed a much higher degree of incompatible splits (i.e., larger loops). Both were largely congruent with the phylogenies estimated using other methods (Figs. 2 and 3).

A comparison of the two Neighbor Nets revealed that the position of several genotypes was different in the two networks (highlighted in red color). G9 (*L. villosa*) was found among southern GBR genotypes in the *ATPSb*-iII Neighbor-Net, whereas they were found among South-Central Pacific genotypes in the rDNA Neighbor-Net, where they were closely related to those from Vanuatu (compare also with Fig. 2). Similarly, G46 (Bali) grouped with genotypes from the north-western Pacific in the *ATPSb*-iII Neighbor-Net, but was closest to a second genotype from Bali (G47) in the rDNA Neighbor-Net. Also, the position of G14 (Hook Reef, GBR) was different in both networks.

#### Estimation of migration rates and directions

Migration rates and directions were estimated for two subsets: 1) a three large-population case, where populations from the East coast of Australia, PNG, and the South Pacific were pooled based on geography (Table 5), and a spatially more restricted six population case, also based on geography, containing five populations from Australia plus the one from PNG (Table 6). Populations contained the same specimens as those used for the AMOVA analysis. Very low migration among populations was estimated for subset 1 (*N*_e_m: 0.05 to 0.31, Table 5), but for the more detailed subset 2, higher asymmetrical migration was estimated within the GBR (mean *N*_e_m up to 3.03, Table 6). Outside the GBR, the only population that was estimated to receive more than one effective immigrant per generation was PNG (*N*_e_m up to 1.32 from the Central GBR, Table 6).

**Table 5:** Migration rates among three pooled populations from Papua-New Guinea (PNG), Australia, and the SW Pacific.

	to Australia	to PNG	to the S'Pacific
Australia	Θ 0.01483	0.31 (109.68)	0.05 (16.26)
PNG	0.12 (31.81)	Θ 0.01127	0.05 (16.68)
S'Pacific	0.05 (13.04)	0.05 (16.2)	Θ 0.01126

Θ (Effective population size [*N*_e_] * mutation rate [μ]), migration rates (*M*), and the number of effective migrants per generation (*N*_e_m) among three larger populations in the SW Pacific as estimated by MIGRATE. Populations were pooled: Australia; Papua New Guinea (PNG); Southern Pacific (S'Pacific). For immigration occurring between populations, the upper row is the receiving population and the left column is the broadcasting population. Θ values are given in the diagonal. The first number in the other cells is *N*_e_m, derived by calculating (Θ * *M*)/4, and the numbers in parentheses are migration rates (*M*). The number of alleles used were (*ATPSb*-iII/ITS) for Australia: 116/186; PNG: 26/38; and S'Pacific: 34/46, for a total of 176/270.

**Table 6:** Migration rates among six populations along the Northeast coast of Australia and Papua-New Guinea (PNG).

	to N' GBR	to Cen. GBR	to Cap. GBR	to BNE	to Coral Sea	to PNG
N' GBR	Θ 0.00259	3.03 (598.86)	0.37 (274.15)	0.20 (285.07)	0.37 (320.57)	1.15 (231.89)
Cen. GBR	0.16 (254.62)	Θ 0.02026	0.31 (231.56)	0.20 (276.95)	0.31 (269.76)	1.32 (265.87)
Cap. GBR	0.15 (237.15)	1.61 (317.63)	Θ 0.00537	0.29 (409.75)	0.27 (234.11)	1.09 (219.98)
BNE	0.13 (202.29)	1.33 (261.62)	0.51 (381.39)	Θ 0.00285	0.25 (215.01)	1.02 (205.91)
Coral Sea	0.14 (220.66)	1.46 (288.97)	0.25 (184.13)	0.19 (262.04)	Θ 0.00458	1.21 (243.72)
PNG	0.13 (198.65)	1.45 (285.66)	0.25 (188.54)	0.19 (273.07)	0.28 (248.76)	Θ 0.01982

Θ (Effective population size [*N*_e_] * mutation rate [μ]), migration rates (*M*), and number of effective migrants per generation (*N*_e_m) among six populations along the Northeast Australian coast and Papua New Guinea (PNG), as estimated by MIGRATE. Populations were: N'GBR (Northern Great Barrier Reef [GBR]); Cen. GBR (Central GBR); Cap. GBR (Capricorn Section GBR); BNE (Sunshine Coast & Brisbane); Coral Sea (Osprey, Bougainville and Holmes Reefs); and PNG (Papua New Guinea). For immigration occurring between populations, the upper row is the receiving population and the left column is the broadcasting population. Θ values are given in the diagonal. The first number in the other cells is *N*_e_m, derived by calculating (Θ * *M*)/4, and the numbers in parentheses are migration rates (*M*). The numbers of alleles used were (*ATPSb*-iII/ITS) for N'GBR: 22/23; Cen. GBR: 18/16; Cap. GBR: 42/33; BNE: 16/8; Coral Sea: 26/27; and PNG: 28/20, for a total of 152/127.

## Discussion

In this study, the phylogeographic structure of the widely distributed coral reef sponge *Leucetta chagosensis *was investigated throughout its known Indo-Pacific range, using two unlinked nuclear DNA markers, the rDNA ITS, a fragment of the 28S gene (C2-D2 region), and a novel marker for sponge evolutionary studies, the second intron of the *ATP synthetase beta subunit *gene (*ATPSb*-iII). Deep genetic divergences, substantial phylogeographic structure, and substantial amounts of regional isolation with low amounts of migration among regional populations were uncovered, and were congruent across both loci. Based on the present data, we argue that life history traits (low dispersal capabilities) in combination with historical factors (tectonics, sea level fluctuations) are responsible for the diversification of the study taxon over space and time.

### Connectivity, or lack thereof

A relationship is expected to exist between the amount of genetic differentiation of allopatric marine populations and their dispersal abilities [23], with low dispersing taxa showing higher genetic structuring and a significant correlation between genetic and geographic distance (isolation-by-distance, IBD) compared to those capable of wide dispersal [24]. In this study, genetic variation was significantly structured among and within populations, but no IBD was detected across the whole set of populations. This suggests that, overall, the colonization process of *L. chagosensis *was discontinuous, with founder effects occurring when new populations were established [10], and that the very low migration rates were responsible for the lack of genetic cohesiveness. A weak, but significantly positive correlation between *F*_ST _and geographic distance was detected among populations connected along the South Equatorial Current (SEC), consistent with expectations based on low dispersal capabilities; some populations on the Australian east coast were also highly differentiated despite their close geographic proximity, probably because there was an area of overlap of two of the deepest diverging clades (see below).

Occasional gene flow in a stepping-stone model [25,26], in which dispersal and genetic exchange occur only between adjacent populations, was suggested by the significant negative slopes of the RMA regression of the logarithmic transformed values (distances and *M*) for both the whole set of populations and the ones along the SEC. However, the negative slopes (respectively -0.3451 and -0.4177) were shallower than expected for a strict stepping-stone model (-0.5 for a two-dimensional array, [26]) and might indicate some long-distance dispersal [27]. Unfortunately, low geographic coverage of *L. chagosensis *samples from the South Pacific (e.g. Fiji, Samoa, etc.) prohibited further analysis and inference at this stage.

The low migration rates, below one effective migrant per generation, frequently observed here are less than the minimum required (i.e., 1 *N*_e_m) to compensate for genetic drift, and thwart continued genetic divergence of populations [28]. For the GBR, these values are lower than those reported for GBR invertebrates [29,30]. In a conservation context, where some authors have argued for the use of a threshold of 10 effective migrants per generation as a minimum to counteract local extinction risks [31], the overall low number of migrants encountered here is cause for concern because isolated populations are clearly prone to local extinction. Such a high degree of regional isolation certainly needs to be taken into consideration for the effective design of marine protected areas [1].

**Figure 6:**
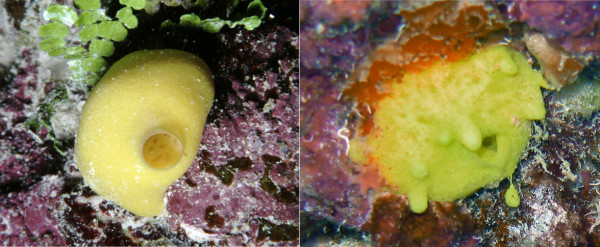
***Leucetta chagosensis *in its natural habitat**. *Leucetta chagosensis *in its natural habitat at Ribbon Reef #10, Great Barrier Reef, February 2006. Left: A small specimen with one central osculum. Right: An individual producing asexual buds separating from the main animal. The size of both specimens is approximately 4 cm.

Results from the present study suggest that the colonization of new distant habitats by *L. chagosensis *and the genetic cohesiveness among them cannot be sustained by the dispersal of sexual propagules alone. Occasional long-distance dispersal, either by asexual fragments [32,33] or by rafting [34] might play a role. To date, there is no direct evidence for long-range dispersal in the studied taxon, but the discovery of a budding specimen of *L. chagosensis *(Fig. 6; G. Wörheide pers. obs. at Ribbon Reef No. 10 in February 2006) provides evidence that asexual dispersal does occur naturally. Such asexual fragments (buds) constitute fully functional mini-sponges that are probably capable of surviving for a considerable amount of time in the plankton (or rafting on pumice, for example) before potentially colonizing distant reef habitats.

### Genealogical patterns

The structure of the rDNA sequence type network, with the two most common sequence types at opposite ends of the network ("star-bursts") connected by a longer branch, is reminiscent of a "dumbbell" shaped network [35], which apparently indicates that two long-separated populations each underwent a (recent) expansion. This interpretation would support a scenario where populations were separated during low sea level stands, and subsequently expanded with rising sea levels from refuges on the Queensland Plateau and the shallower shelf south of the GBR [15]. This hypothesis, originally formulated by Davies [36], is also supported by a number of other genetic studies on fish and corals (e.g. [37-39]). However, such a vicariance scenario is complicated by the intermediate allopatric populations in the rDNA network, and was not supported by a recent study of calcareous sponges from the same calcarean family Leucettidae, *Pericharax heteroraphis*, using the same molecular markers (ITS rDNA, *ATPSb*-iII) [40]. Here, Bentlage and Wörheide observed much less variation in both markers and no phylogeographic (genealogical) structure on the GBR, and attributed this to the retention of ancestral ITS rDNA polymorphisms and a relatively recent expansion after a population bottleneck. This suggests considerable amounts of idiosyncrasy in each species' (demographic) history.

Using the rDNA internal transcribed spacers (ITS) for phylogenetic inferences poses some difficulties. The internal transcribed spacers (ITS) separate the 18S and 28S rDNA genes in the tandemly repeated rDNA cistron [41]. While variation among the multiple ITS copies is normally homogenized by a process called 'concerted evolution' [42], intragenomic polymorphisms (IGP) [21] do occur, e.g., if concerted evolution is slow [43]. The occurrence of potentially paralogous ITS copies can then confound phylogenetic inferences [21,44]. The few rDNA IGPs detected in this study could all be resolved into two different sequence types, and were always found to be closely related and clade-specific, most likely representing orthologs. Nonetheless, reconstruction of sequence types from rDNA IGPs by haplotype inference should always be preceded by subcloning of rDNA amplicons of several individuals to check the extent of intragenomic polymorphism [21]. We consider the risks of analysing paralogous rDNA sequence types to be minimal in this case, but the inclusion of additional and independent loci is necessary to untangle the true demographic history from locus-specific forces because only genealogical concordance across multiple unlinked loci can elucidate whether phylogeographic breaks are caused by stochasticity or by real barriers to gene flow [45].

To this end, our analysis of *ATPSb*-iII sequences provided pivotal insight. This new marker showed about five-fold higher substitution rates than the rDNA, and higher phylogeographic resolution was achieved with congruent deeper coalescent patterns across loci. Based on the data presented here, we argue that the high concordance between the two loci is due to population substructuring and putative reproductive isolation, and not to the linked inheritance of both loci (for which no data currently exist).

The large divergence of the reciprocally monophyletic genotypes from the Philippines, with their divergence especially pronounced among the intron alleles, points to a long-standing isolation of the Philippine population. The Philippine islands are an area of complex island-geomorphology that have undergone complicated tectonics, with some islands on crustal fragments originating far south-east of their present position [46]. Several of today's islands have emerged since the late Miocene (about 10 million years ago), and the sampled area at the northern reef of Bohol probably belonged to Proto-Mindanao during the Pliocene [47]. Initial colonization could have happened by long-distance dispersal soon after reef habitats were established. This could then have been followed by persistence when subsequent changes in shoreline constellations due to cycling sea levels reinforced the genetic isolation of this region [29], resulting in the deeply divergent and reciprocally monophyletic clade discovered here. Such apparent founder speciation has been previously described in the terrestrial fauna of the Philippines [47], but might be more widespread in marine biota than previously appreciated [10]. This could also be responsible for generating and maintaining the other reciprocally monophyletic populations discovered here. However, for more in-depth interpretations of the remarkable divergence of the Philippine population, a broader geographic coverage of the whole island group would be required.

Substantial amounts of local endemism (reciprocal monophyly) were also uncovered, pointing to considerable amounts of regional isolation similar to what has been found in some other reef invertebrates [7]. This is also consistent with expectations based on low dispersal capabilities. However, some populations were found to be para- or polyphyletic. Such a pattern of population poly- or paraphyly may be caused by either incomplete lineage sorting or recurrent (recent and historic) gene flow among previously separated clades [35], or both of these factors. These two processes of shared alleles among distinct clades are hard to distinguish on the basis of genetic data alone [48]. Avise [35] proposed that deep coalescence of polyphyletically distributed lineages would point to incomplete lineage sorting, whereas shallow genealogical patterns would be caused by recent gene flow. For *L. chagosensis*, all polyphyletic populations (e.g. PNG, GBR) showed deep coalescence, as expected for a scenario of incomplete lineage sorting. However, the previous differentiation of geographically restricted reciprocally monophyletic lineages with subsequent re-dispersal into each others' ranges are equally likely to have caused the apparent overlap in geographic distribution of the two deepest diverging clades in the area of PNG and East Australia. Based on the presence of putative hybrids (see below), we favor the latter scenario. However, more extensive geographic sampling from those critical regions would be beneficial to allow additional conclusions.

Under the phylogenetic species concept [49,50], all reciprocally monophyletic groups uncovered here would represent different recognizable taxa and ESUs (Evolutionary Significant Units [51]). The degree of reproductive isolation among those ESUs cannot be discerned at present, i.e., whether they are from a single, still-interbreeding widespread species, or instead constitute a reproductively isolated sibling (and morphologically cryptic) species. Support for the latter came from the near-fixation of alternative *ATPSb*-iII alleles in some populations, e.g., between the populations from Brisbane and Polynesia. The occurrence of a different combination of *ATPSb*-iII alleles and rDNA sequence types in some individuals, uncovered by their different clade-affiliations, is an indication of occurrences of hybridization and incomplete reproductive isolation. One of those hybrids is *L. villosa*, was described as a closely related sister-species of *L. chagosensis *based solely on morphological data [14]. This is evidence that those hybrids are also manifested as (morphologically) distinguishable phenotypes.

### The temporal aspect

Resolving the temporal frame of clade divergences in *L. chagosensis *remains a challenge due to the paucity of unequivocally identifiable fossil remains. We also lack information about mutation rates in this taxon. Only from the study of Wörheide et al. [21], who analysed divergences in rDNA ITS sequences in several reciprocally monophyletic populations of *Prosuberites laughlini *(Demospongiae: Hadromerida) across the Isthmus of Panama, can we derive a mutation rate of roughly 1% ITS rDNA sequence divergence per million years for this demosponge. While the validity of applying this mutation rate estimate to a phylogenetically distant taxon is untested, it does currently represent the only option for a rough estimation of divergence times among *L. chagosensis *clades. Thus, the deepest divergences among *L. chagosensis *ITS rDNA sequence types would have happened about 2 million years ago in the late Pliocene, and subsequent divergences would have occurred in the Pleistocene (see Table 1). The late Pliocene, an era with fluctuating sea levels [52], and the resulting differences in shoreline distributions and current (dispersal) patterns, has been estimated to be the time of vicariant speciation in a number of marine taxa, as has the Pleistocene [29].

## Conclusion

Deep phylogeographic structure was uncovered in this study, congruent across the two nuclear markers used. There was low gene flow among most regional populations, some of which showed isolation-by-distance effects. Overall, dispersal was indicated in a stepping-stone model with some long-distance genetic exchange – all consistent with expectations from the allegedly low dispersal capability of the taxon. Reciprocally monophyletic populations have long been isolated and constitute putative sibling species, but the degree of reproductive isolation between para- and polyphyletic populations remains to be investigated in more detail. Hybrids displaying mixed genotypes from deeply divergent clades were rare, but one (*L. villosa*) was morphologically distinguishable. *L. chagosensis *might therefore be regarded as a complex of closely related (cryptic) species rather than a single widespread species.

However, we cannot conclusively determine at present whether the observed structure was generated solely by vicariance resulting from glacio-eustatic changes during the late Pliocene and Pleistocene, or was simply a result of general dispersal limitation with stochastic long-distance dispersal and founder speciation. However, both processes supposedly contributed with varying degrees to the generation and/or maintenance of the observed phylogeographic structure in each region.

The low migration rates observed here are not sufficient to counteract the continued genetic divergence and regional isolation of populations. Geographically restricted and deeply divergent genealogical lineages of taxa with low dispersal capabilities are therefore prone to local extinction, since immigrants from more viable, distant habitats cannot easily replenish populations depleted of genetic diversity. These results highlight the need for comprehensive biodiversity estimates (i.e., based on true genetic diversity) when considering an effective design and implementation of marine reserves, in order to conserve the complete diversity to promote reef resilience.

## Methods

### Sample collection

*Leucetta chagosensis *specimens were collected by SCUBA diving on subtidal reef slopes. Specimens from the East Coast of Australia, the Coral Sea, Vanuatu, the Red Sea and Japan were collected by Gert Wörheide (GW); other specimens were received from various colleagues. Due to this fact, and because this taxon can be quite rare (e.g. in the Gulf of Aqaba and the Red Sea), sample sizes were limited in some localities, varying from two in Fiji to 10 in the Philippines individuals per site. Voucher sample numbers and localities are given in Additional file 1. Immediately after collection on deck, macroinvertebrate commensals were carefully removed, if present, and parts of the choanosome were cut into small pieces. Sometimes, small specimens were preserved whole. Samples were preserved in >90% ethanol or in silica-gel after being cut into small pieces, and were stored at -20°C or room temperature until extraction (see also [15,53]). The external surface was avoided to minimize potential contamination. Voucher samples have been deposited at the Queensland Museum, Brisbane, Australia (indicated by the prefix "QMG" in Additional Table 1 see Additional File 1) or are held by G. Wörheide.

### DNA extraction and PCR amplification

Genomic DNA was extracted from the choanosomal tissue using the DNeasy-Tissue Kit (Qiagen) according to the manufacturer's instructions. Internal transcribed spacer (ITS) and partial 28S (C2-D2 region [54]) rDNA sequences were generated according to previously published protocols [15,21,55].

The second intron of the *ATP synthetase beta subunit *gene (*ATPSb*-iII) was amplified using the following strategy. Initially, degenerate primers (ATPSbf1: 5'-CGT GAG GGH AAY GAT TTH TAC CAT GAG ATG AT-3'; ATPSbr1: 5'-CGG GCA CGG GCR CCD GGN GGT TCG TTC AT-3'; [56]) were used for PCR amplification of four samples from disparate geographic areas (Red Sea, Taiwan, GBR, Tuamotu). BLAST searches [57] were run to confirm that the amplified sequences were of poriferan origin. Exon/intron boundaries were determined by comparison with ATPS beta gene sequences from Genbank. The intron was determined to start about 2.3 kb downstream from the 5'-end of Exon 1 in *Drosophila melanogaster *(Position 3416; GenBank accession no. X86015). From an initial alignment, nested species-specific primers were designed (ATPSbf2: 5'-TTG TCT TGG ACA AGG AGG GG-3'; ATPSbr2: 5'-TCG TTC ATT TGA CCG TAC AC-3'), which were still located in the exon but were about 10–15 bp closer to the exon/intron boundary. These primers were then used for subsequent PCR amplification of the larger data set.

PCR reactions consisted of 2 mM MgCl_2_, 16 mM (NH_4_)_2_SO_4_, 67 mM Tris-Cl (pH 8.8 at 25°C), and 0.01% Tween-20. Each 25 *μ*l reaction included 1.0 *μ*l of DNeasy-extracted DNA of various concentration, 0.625 μl dNTPs (10 mM each), 1.5 mM of each primer, and 0.25 U Bio-*Taq *DNA polymerase (Bioline, Luckenwalde). PCR cycling conditions included an initial denaturation of 2 min at 94°C; 38 cycles of 94°C for 20 s, 54°C for 60 s, and 72°C for 50 s; and a final extension step at 72°C for 10 min. Amplicons were purified from Agarose gels using a silica-based method [58]. PCR products were sequenced directly with ABI BigDye Terminator Cycle Sequencing (Version 3.1) on an ABI 3100, and some PCR products were subcloned into pGEM-T (Promega) according to the manufacturer's instructions. A minimum of three positive clones were sequenced in both directions using M13 vector primers to determine if more than two alleles were present, indicating paralogous copies. Amplicons were directly sequenced as above. Because the specific primers were very close to the exon/intron boundaries, the first 42 bases of the 5'- end and the first 53 bases of the 3'- end of the intron were not included in the final alignment.

### Sequence assembly and alignment

Double-stranded sequences of novel sequences generated in this study were assembled with CodonCode Aligner [59], automatically aligned using ClustalW [60], and manually inspected and optimized using Se-Al v2.0a11 [61]. Existing rDNA ITS sequence types of *Leucetta chagosensis *from previous studies [15,21] (Genbank accession nos. AF458852–AF458870) were added to the alignment. Polymorphic sites in heterozygotes were detected using the 'find mutations' option in the CodonCode Aligner, and coded using IUPAC [62] ambiguous DNA characters. Alleles of heterozygotes that showed no length variation within individuals were resolved manually using a parsimony approach, in an attempt to minimize the number of alleles observed in the data set [63]. Intragenomic polymorphisms in rDNA were resolved into two different sequence types per individual using the same approach, primarily to enable estimation of migration rates in MIGRATE (see below). Due to the low intraspecific diversity of the C2-D2 fragment, rDNA alignments were concatenated for subsequent phylogeographic analyses. All sequences from this study were submitted to the EMBL Nucleotide Sequence Database and can be accessed under the following accession numbers: [EMBL: AM850255–AM850677].

### Intron heterozygous indel detection

We attempted to resolve length variant heterozygotes (LVH), which often cause problems when analyzing intron sequences [64], using Single-Stranded Conformational Polymorphism (SSCP) analyses [65], as described in [40]. However, the intron was too long for alleles to be resolved using this method. Therefore, to obtain an overview of the distribution of LVH alleles on the estimated phylogenies, i.e. whether one heterozygote individual harbours alleles distributed in different larger clades on the estimated phylogeny, amplicons of several such heterozygotes from each larger clade were subcloned as above, sequenced, and both alleles were included in the alignment. Due to restricted resources, it was not possible to subclone and sequence both alleles of all heterozygous individuals.

Indels and minisatellite repeats were treated as missing data, and were not recoded for phylogenetic analyses because they represented autapomorphies for regional populations, and as such did not contribute to resolving phylogeographic structure.

### Phylogenetic analyses

All sequences were assembled and aligned as described above. Uncorrected p-distances were calculated using Paup*4 [66]. Nucleotide diversities and GC contents were calculated, and tests of neutrality using Tajima's D [67] were carried out in DNAsp v4.1 [68]. Nuclear loci are subject to recombination, potentially confounding phylogeny estimation [69]. Consequently, recombination rates and events were estimated using RDP 2.0 [70], employing the default options with all methods implemented in the program suite (RDP, GENECONV, Bootscan, Chimera, SciScan). The program MODELTEST version 3.7 [71] was used to find the model of DNA substitution that best fit the rDNA and intron data. The best-fit model selected for each sequenced region by hierarchical Akaike information criterion (AIC) [72] (ITS rDNA: TIM+G, partial 28S rDNA: HKY+I, intron: TrN+G) was used for a subsequent Bayesian phylogeny inference (BI) using MrBayes 3.1.2 [73] with default priors and mixed models.

Phylogenies were estimated from the combined ITS/partial 28S rDNA alignment, the intron alignment, and a combined ITS/28S/intron alignment containing only specimens from which all three fragments could be obtained. For the latter, consensus sequences of *ATPSb*-iII alleles were used and concatenated with both rDNA fragments into one alignment. Alignments were collapsed to contain only unique sequence types/alleles in COLLAPSE 1.2 [74] for phylogeny estimation, and intron sequences were additionally analyzed using the full number of detected alleles.

Three independent runs with one cold and seven heated Markov chains each per analysis were performed simultaneously in MrBayes 3.1.2 until the average standard deviation of split frequencies between the three runs dropped below 0.005 (lowered from the default value of 0.01 to improve chain convergence). Analyses were carried out with the MPI-enabled parallel version of MrBayes [75] on a 64-node Linux-cluster at the Gesellschaft für wissenschaftliche Datenverarbeitung Göttingen (GWDG), using one processor for each of the 24 Markov chains per analysis. Batch files are available upon request. All MrBayes analyses were run at least twice to check for the consistency of results. The second analysis was allowed to run longer without a stop value, until the wall time of 48 hours was reached, to check if convergence of chains could be further improved. Trees were sampled every 1000^th ^cycle, with a burn-in of 25% of sampled trees. The appropriateness of burn-in values was determined after the graphical display of likelihood values, and the convergence of chains was evaluated using AWTY [76]. The remaining trees after burn-in were used to generate a 50% majority rule consensus tree, where posterior probabilities for internal branches were indicated by their sample frequency.

For comparison, Maximum Likelihood bootstrap analyses were conducted with GARLI 0.94 [77] using a heuristic search with the default option, i.e. under the GTR model of nucleotide substitution, with gamma distributed rate heterogeneity, a proportion of invariant sites, and 100 bootstrap replicates. Phylogenies estimated from the intron-only and combined rDNA/intron data set were rooted with the Indian Ocean clades, as they represent a different biogeographic region (e.g. [78]). Outgroup rooting with intron sequences from a closely related species was not possible because such sequences were unalignable due to high evolutionary rates, and because the same intron in *Pericharax heteroraphis*, for example, is about 60% shorter [40].

Due to the low divergence of rDNA sequences, we estimated a statistical parsimony sequence type network [79] using the program TCS 1.21 [80] with the default options. The maximum number of mutational steps that constitute a parsimonious connection between two sequence types was calculated with 95% confidence. An attempt at constructing a nested clade design [81] was not successful because no unambiguous nested design could be constructed due to a central loop that could not be resolved with confidence.

To obtain an overview of the level of ambiguity and phylogeographic congruency within and among loci and to visually explore the different signals contained in the data, the concatenated alignment was divided into rDNA and *ATPSb*-iII partitions. Each partition contained samples from 92 specimens from which both loci could be sequenced. Both loci were analyzed separately using Neighbor-Net [82], as implemented in SplitsTree4 [83], using uncorrected p-distances.

In brief, Neighbor-Net is a distance-based method that provides an effective tool to visualize and detect conflicting signals or alternative phylogenetic histories. First, a collection of weighted splits is constructed from a distance matrix, and then these splits are represented using a splits graph, where a totally compatible collection of splits would be precisely represented as a tree, but incompatible splits as cycles or boxes. Such incompatibilities might represent, among other things, hybridization, recombination, gene duplication, or in general, conflicting signals in the data. In general, the more (and larger) cycles/boxes connecting operational taxonomic units (OTUs) in a split graph, the more incompatibilities of splits and incongruences exist in the data.

### Population genetic analyses

Analysis of molecular variance (AMOVA) [84] was conducted to estimate the significance of population structure at several hierarchical levels. Pairwise fixation indices (*F*_st_) [85] and their significance were calculated for intron alleles only to estimate population differentiation because true heterozygotes cannot be distinguished from intragenomic variation in the rDNA cistron (see [86]), however, an AMOVA was carried out using the rDNA sequence types. Calculations were carried out in ARLEQUIN 3.1 [87] using 10,100 random permutations for significance tests. All analyses were run at least twice to check for the consistency of results. Two sets of analyses were run for each data set separately, because Arlequin does not allow for unequal sample sizes among loci. First, sample localities were pooled into 15 geographic populations. Pooling of sample localities into populations on the Great Barrier Reef (GBR) followed the sections of the Great Barrier Reef Marine Park: N'GBR, Central GBR, Capricorn (GBR), Brisbane, Queensland Plateau; Taiwan, Guam, Okinawa, Philippines, Indonesia; Maldives, Red Sea; and PNG, Samoa/Fiji/Vanuatu, Polynesia. The 15 populations were grouped into four regional groups (separated by semicolons in the previous list: Australia, NW Pacific, S'Pacific, and Indian Ocean). Due to low sample sizes from some archipelagos in the SW Pacific (Fiji, Samoa) in the intron data set, those localities were pooled with samples from Vanuatu as one population in order to increase the statistical power. To enable comparison between the two loci, the same geographic population structure was employed for the rDNA data set, where more sequences were available from those archipelagos. A second analysis included only eight populations from the SW Pacific (Northern GBR, Central GBR, Capricorn, Brisbane, Queensland Plateau, PNG, Samoa/Fiji/Vanuatu, and Polynesia), grouped into two groups (Australia, S'Pacific), where sampling was more comprehensive both in terms of geography and sample sizes. A Mantel test was carried out using the Isolation by Distance Web Service [88] to test for correlations between spatial and genetic (*F*_st_) distances [89], also using gene flow *M *calculated as (1/*F*_ST_-1)/4 [26]. The significance of the slope of the reduced major axis (RMA) regression was assessed by 30,000 randomizations.

Because of the acknowledged difficulties of using *F*_ST_'s to estimate gene flow [90], we also took a coalescent-based approach to estimate the parameters of populations [91], such as theta (θ = 4*N*_e _μ; where *N*_e _is the effective number of individuals and μ is the mutation rate in mutations per generation), migration rates (*M*), and the number of effective migrants per generation (*N*_e_m). We used the coalescent-based Markov-Chain Monte Carlo method implemented in MIGRATE 2.1.7, which explicitly takes into account historical processes and asymmetrical migration/gene flow [91]. The parallelized version of MIGRATE was compiled to run on the LINUX-cluster of the GWDG (see above), requesting several processors for each run depending on the numbers of replicates. Several independent runs were conducted to check for convergence, the consistency of results, and the shape of posterior distributions. The Bayesian search strategy was optimized for the following settings (parm files available on request): recorded genealogies [a]: 100,000; increment (record every x genealogy [b]: 500; and the number of concurrent chains [c]: 4, resulting in 2*10^8 ^visited (sampled) genealogies [a*b*c] with 10,000 discarded trees per chain (burn-in). For MIGRATE analyses, each diploid sponge individual was represented by two alleles/sequence types in the input file, whether it was homozygous or heterozygous. rDNA sequences showing IGPs were resolved into two sequence types using the parsimony approach outlined above, since MIGRATE does not allow ambiguously (IUPAC) coded nucleotide sites.

Due to the large geographic distances between some populations without intermediate sample localities, (e.g. Red Sea-Maldives-IWP) and the low sample sizes of some, we focussed our attention on estimating the migration rates among populations along the East-Australian coast, Papua New Guinea, and the southern Pacific only.

## Authors' contributions

GW conceived and designed the study, analysed and interpreted the data, and drafted and revised the manuscript. LSE carried out part of the molecular genetic work (rDNA) and contributed to data analysis and interpretation as well the manuscript revision. LM carried out part of the molecular work (*ATPSb*-iII) and revised the manuscript. All authors read and approved the final manuscript.

## Supplementary Material

Additional file 1 Details of samples, including allele/sequence type/genotype designation and latitude/longitude data, as well as EMBL Accession numbers for each fragment used. Novel sequences generated in this study in bold. Note several sheets in file. Click here for file

Additional file 2 1) Amino acid alignment of three sponge *ATP Synthetase beta subunit *sequences from Genbank and the sequence of *Leucetta chagosensis *(Specimen No. QMG 316175); 2) corresponding nucleotide alignment. Click here for file

Additional file 3 Bayesian phylogeny of 89 *ATPSb*-iII alleles with all posterior probability values from Bayesian analysis and Maximum Likelihood non-parametric bootstrap proportions indicated at branches. Click here for file

Additional file 4 Bayesian phylogeny of 92 unique genotypes from the concatenated rDNA and *ATPSb*-iII alignment with all posterior probability values from Bayesian analysis and Maximum Likelihood non-parametric bootstrap proportions indicated at branches. Click here for file

Additional file 5 Neighbor-Net analysis of the different partitions (rDNA and *ATPSb*-iII) and the concatenated alignment of 92 unique genotypes found among the 105 individuals from which sequences of both loci could be obtained. Click here for file
