# Supplementary material for: Deep genetic divergences among Indo-Pacific populations of the coral reef sponge Leucetta chagosensis (Leucettidae): Founder effects, vicariance, or both?
Source: BMC Evol Biol. 2008 Jan 26;8:24. doi: 10.1186/1471-2148-8-24 (PMC2267160; doi:10.1186/1471-2148-8-24)
Supplement: Additional file 2 — 1) Amino acid alignment of three sponge ATP Synthetase beta subunit sequences from Genbank and the sequence of Leucetta chagosensis (Specimen No. QMG 316175); 2) corresponding nucleotide alignment. [file 1471-2148-8-24-S2.pdf]

```
DQ087473Microciona      1 PKTLGRIINVIGEPIDERGPIGEEMQAAIHADAPEFVDMNVKQEILVTGIKVVDLLAPYA
DQ087459Sycon           1 VETLGRIMNVIGEPIDERGPINAKATAPIHAEAPEFVDMSVEQEILETGIKVVDLLAPYA
DQ087466Leucosolenia    1 PETLGRIMNVIGEPIDERGPIDAKASAPIHAEAPEFVDMSVEQEILETGIKVVDLLAPYA
316175_Klon3_Exon       1 ------------------------------------------------------------

DQ087473Microciona     61 KGGKIGLFGGAGVGKTVVIMELINNVAKAHGGCSVFAGVGERTREGNDLYHEMIQGGVIS
DQ087459Sycon          61 KGGKIGLFGGAGVGKTVLIMELINNVAKAHGGYSVFAGVGERTREGNDLYHEMIVGGVIV
DQ087466Leucosolenia   61 KGGKIGLFGGAGVGKTVLIMELINNVAKAHGGYSVFAGVGERTREGNDLYHEMIVGGVIV
316175_Klon3_Exon       1 -------------------------------------------REGNDFYHEMITGGVIV

                                 *
DQ087473Microciona    121 LKDDSSK/VALVYGQMNEPPGARARVALTGLTVAEYFRDQEGQDVLLFIDNIFRFTQAGS
DQ087459Sycon         121 LDKPGSK/VALVYGQMNEPPGARARVALTGLTVAEYFRDKEGQDVLLFIDNIFRFTQAGS
DQ087466Leucosolenia  121 LDKPGSK/VSLVYGQMNEPPGARARVALTGLTVAEYFRDKEGQDVLLFIDNIFRFTQAGS
316175_Klon3_Exon      18 LDKEGSK/VSLVYGQMNEPPGARARI-----------------------------------

DQ087473Microciona    180 EVSALLGRIPLLWATSQTLATDMGTMQERITTTQKGSITSVQAVFVPADDLTDPAPATTF
DQ087459Sycon         180 EVSALLGRIPSAVGYQPTLATDMGSMQERITTTKEGSITSVQAIYVPADDLTDPAPATTF
DQ087466Leucosolenia  180 EVSALLGRIPSAVGYQPTLATDMGSMQERITTP-EGSITSVQAIYVPADDLTDPAPATTF
316175_Klon3_Exon         ------------------------------------------------------------

DQ087473Microciona    240 AHLDATTVLSRAISELGIYPAVDPLDSTSRIMDPNIVGAEHYNVARGVQKILQDYRSLQD
DQ087459Sycon         240 AHLDATTVLSRGIAELGIYPAVDPLDSTSRILDPNIVGQEHYSIARSVQKILQDYKSLQD
DQ087466Leucosolenia  239 AHLDATTVLSRGIAELGIYPAVDPLDSTSRILDPNTVGMEHYTIARSVQKILQDYKSLQD
316175_Klon3_Exon         ------------------------------------------------------------

DQ087473Microciona    300 IIAILGMDELSEDDKLTVARARKIQRFLSQPFQVAEVFTGKEGKLVSLENTISGFQKIIN
DQ087459Sycon         300 IIAILGMDELSEEDKLTVARARKIQRFLSQPFQVAEVFTGKPGKFVSLEQTIKGFTSILN
DQ087466Leucosolenia  299 IIAILGMDELSEEDKLTVSRARKIQRFLSQPFQVAEVFTGKPGKFV--------------
316175_Klon3_Exon         ------------------------------------------------------------

DQ087473Microciona    360 GELDDIPE
DQ087459Sycon         360 GEHDHIPL
DQ087466Leucosolenia      --------
316175_Klon3_Exon         --------
```

Additional Figure 1: Amino acid alignment of three sponge ATP Synthetase beta subunit sequences from Genbank (accession numbers DQ087473, DQ087459, DQ087466) and the sequence of *Leucetta chagosensis* (Specimen No. QMG 316175). Location of intron is indicated by a forward slash and * above the site. Black alignment background indicates conserved alignment positions, grey backgound similar chemical properties, white background variable positions with dissimilar amino acids.

```
DQ087473Microciona      1 GAGAGAACACGTGAGGGAAACGATCTTTATCACGAGATGATCCAGGGAGGTGTCATCAGT
DQ087459Sycon           1 GAGCGTACCCGTGAGGGTAACGATTTGTACCATGAAATGATCGTTGGAGGCGTCATCGTC
DQ087466Leucosolenia    1 GAGCGTACGCGTGAAGGTAACGACTTGTATCACGAAATGATCGTCGGTGGTGTCATCGTC
316175_Klon3_Exon       1 ---------CGTGAGGGCAATGATTTTTACCATGAGATGATCACTGGTGGCGTTATTGTC
316175_Klon3_Full       1 ---------CGTGAGGGCAATGATTTTTACCATGAGATGATCACTGGTGGCGTTATTGTC

DQ087473Microciona     61 CTGAAGGATGACAGTTCTAAG----------~----------GTGGCGCTGGTGTATGGA
DQ087459Sycon          61 CTGGATAAGCCTGGATCGAAG----------~----------GTCGCTCTTGTGTACGGT
DQ087466Leucosolenia   61 CTGGACAAGCCAGGTTCCAAG----------~----------GTATCCCTTGTGTACGGT
316175_Klon3_Exon      52 TTGGACAAGGAGGGGTCGAAG----------~----------GTATCGCTGGTGTACGGT
316175_Klon3_Full      52 TTGGACAAGGAGGGGTCGAAGGTGAGCAAGT~GTGTTTCAAGGTATCGCTGGTGTACGGT
                                               |      Intron       |

DQ087473Microciona    100 CAGATGAACGAGCCCCCAGGTGCCCGCGCACGTGTGGCCCTGACT
DQ087459Sycon         100 CAGATGAACGAGCCCCCGGGAGCCCGTGCCCGTGTCGCTTTGACC
DQ087466Leucosolenia  100 CAGATGAACGAGCCCCCGGGAGCCCGTGCCCGTGTTGCCCTTACC
316175_Klon3_Exon      91 CAAATGAACGAACCACCCGGCGCCCGTGCCCGAATC---------
316175_Klon3_Full     111 CAAATGAACGAACCACCCGGCGCCCGTGCCCGAATC---------
```

Additional Figure 2: Nucleotide alignment of three sponge ATP Synthetase beta subunit sequences from Genbank (accession numbers DQ087473, DQ087459, DQ087466) and the sequence of *Leucetta chagosensis* (Specimen No. QMG 316175), the upper one contains only the exon sequence, the lower one (..._full) also 5' and 3' fragments of the phase-0 intron that follows the GT-AG rule. The missing central part of the intron is indicated by "~". Black alignment background indicates conserved alignment positions, grey and white background to indicate variable positions.
