# Supplementary figures and images for: Deep genetic divergences among Indo-Pacific populations of the coral reef sponge Leucetta chagosensis (Leucettidae): Founder effects, vicariance, or both?
Source: BMC Evol Biol. 2008 Jan 26;8:24. doi: 10.1186/1471-2148-8-24 (PMC2267160; doi:10.1186/1471-2148-8-24)

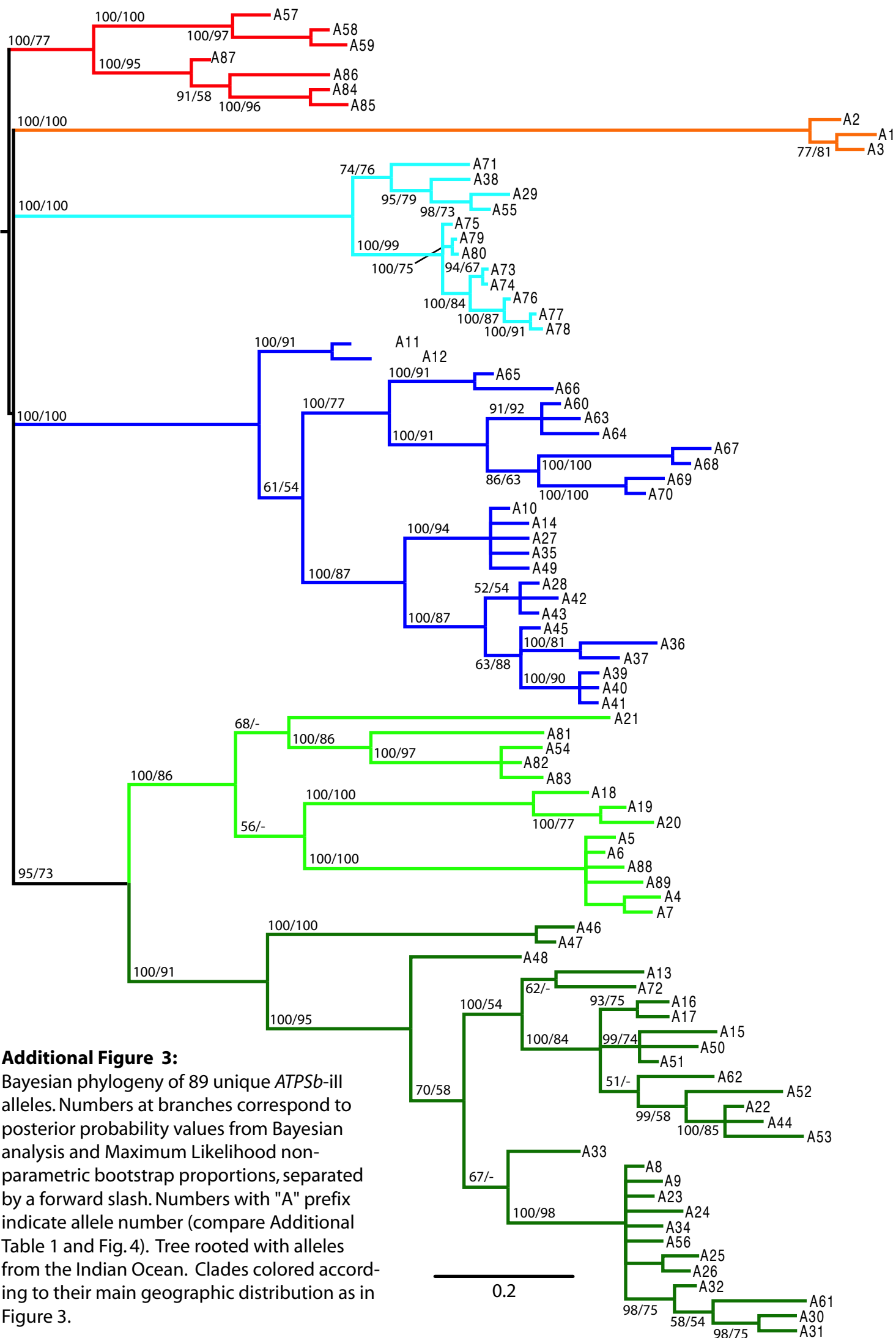

Supplement: Additional file 3 — Bayesian phylogeny of 89 ATPSb-iII alleles with all posterior probability values from Bayesian analysis and Maximum Likelihood non-parametric bootstrap proportions indicated at branches. [file 1471-2148-8-24-S3.pdf]

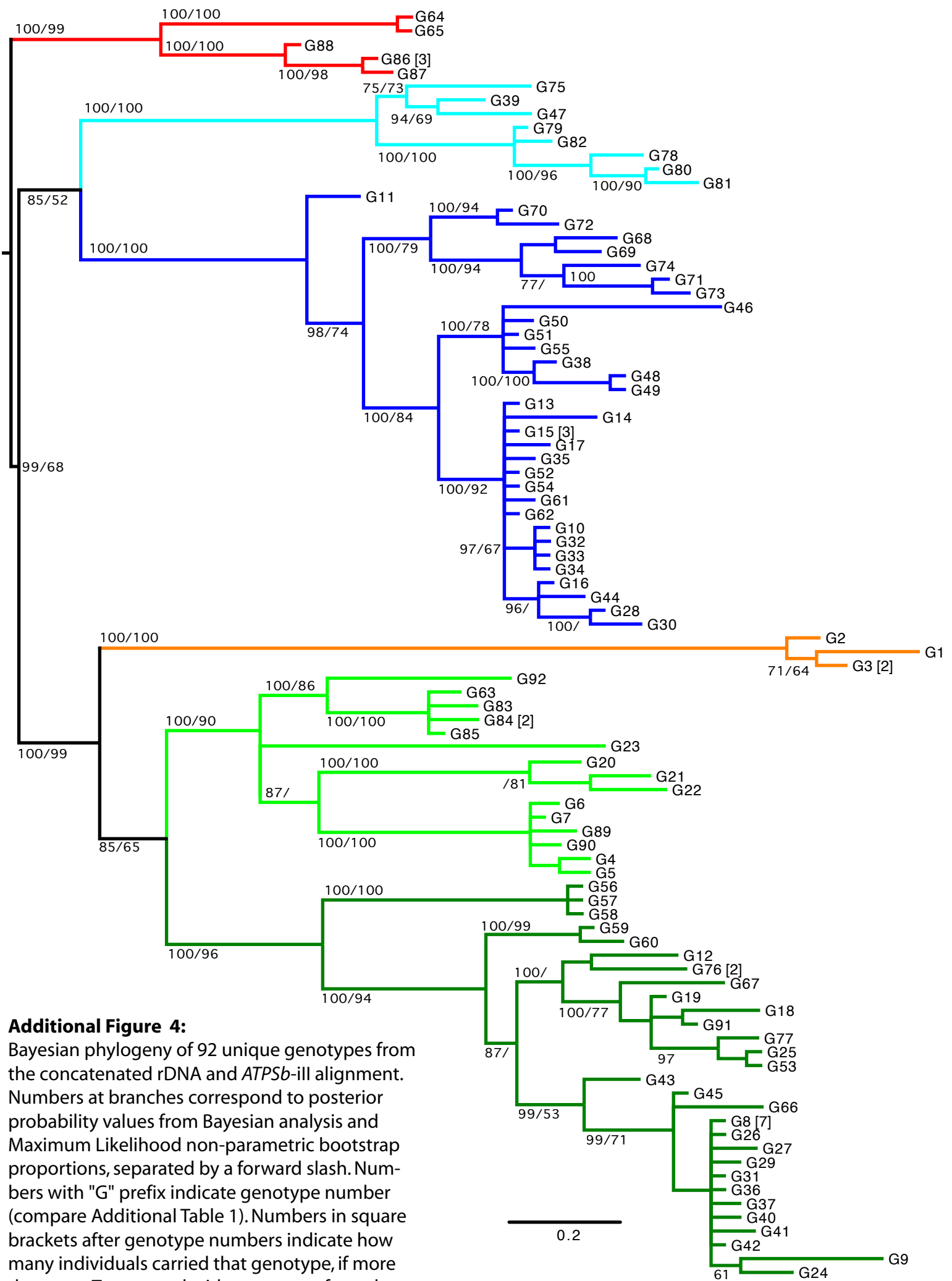

Supplement: Additional file 4 — Bayesian phylogeny of 92 unique genotypes from the concatenated rDNA and ATPSb-iII alignment with all posterior probability values from Bayesian analysis and Maximum Likelihood non-parametric bootstrap proportions indicated at branches. [file 1471-2148-8-24-S4.pdf]
