# Supplementary material for: Deep genetic divergences among Indo-Pacific populations of the coral reef sponge Leucetta chagosensis (Leucettidae): Founder effects, vicariance, or both?
Source: BMC Evol Biol. 2008 Jan 26;8:24. doi: 10.1186/1471-2148-8-24 (PMC2267160; doi:10.1186/1471-2148-8-24)
Supplement: Additional file 5 — Neighbor-Net analysis of the different partitions (rDNA and ATPSb-iII) and the concatenated alignment of 92 unique genotypes found among the 105 individuals from which sequences of both loci could be obtained. [file 1471-2148-8-24-S5.pdf]

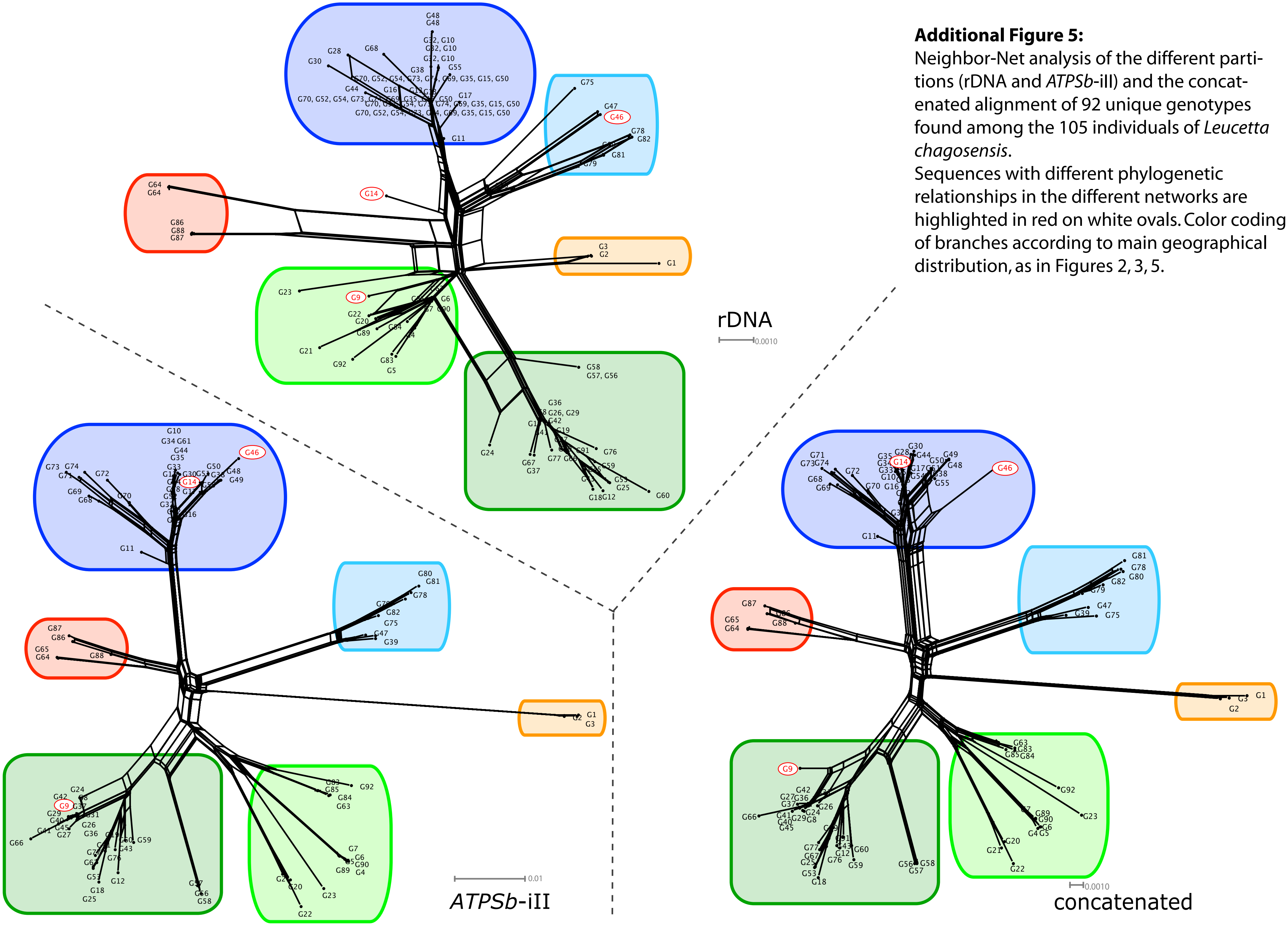

Additional Figure 5:
Neighbor-Net analysis of the different partitions (rDNA and *ATPSb*-iII) and the concatenated alignment of 92 unique genotypes found among the 105 individuals of *Leucetta chagosensis*.
Sequences with different phylogenetic relationships in the different networks are highlighted in red on white ovals. Color coding of branches according to main geographical distribution, as in Figures 2, 3, 5.
